# Trends of potentially inappropriate prescribing among older outpatients in China between 2015–21

**DOI:** 10.7189/jogh.15.04190

**Published:** 2025-06-20

**Authors:** Fangyuan Tian, Zhaoyan Chen, Ying Zhang, Qiyi Feng

**Affiliations:** 1 Department of Pharmacy, National Clinical Research Centre for Geriatrics, West China Hospital, Sichuan University, Chengdu, China; 2 Department of Epidemiology and Health Statistics, West China School of Public Health and West China Fourth Hospital, Sichuan University, Chengdu, China; 3 Precision Medicine Research Centre, Sichuan Provincial Key Laboratory of Precision Medicine and National Clinical Research Centre for Geriatrics, West China Hospital, Sichuan University, Chengdu, China

## Abstract

**Background:**

Potentially inappropriate medication (PIM) remains a significant global concern due to its association with adverse drug events in older adults. The prevalence of potentially inappropriate prescriptions (PIPs) varies considerably across different countries. However, there is a lack of evidence using standardised Chinese national criteria to explore variations in prescribing trends. We aimed to evaluate the trends in the prevalence of PIP based on these criteria.

**Methods:**

In this descriptive epidemiological study, we utilised prescription data from older outpatients across 59 hospitals in six major geographic regions of China. We analysed the prevalence of PIP overall, as well as the prevalence of PIP caused by varying numbers of PIMs and high-risk PIMs (clopidogrel, estazolam, zolpidem, sliding-scale insulin, alprazolam) for the period between 2015–21. We calculated the average annual percent change (AAPC) using joinpoint regression to examine temporal trends.

**Results:**

A total of 982 605 prescriptions for older outpatients between 2015–21 were included in the analysis. The prevalence of PIP related to multiple PIMs increased from 4.70% to 6.09% (AAPC = 4.45). Notably, the prevalence of PIP associated with estazolam (AAPC = 3.71), zolpidem (AAPC = 13.51), and alprazolam (AAPC = 8.86) demonstrated a steady upward trend, while the prevalence of PIP linked to clopidogrel (AAPC = –4.70) consistently declined.

**Conclusions:**

We observed an increasing trend in PIP associated with sedative-hypnotic medications. It demands heightened attention in subsequent use to ensure drug safety.

By 2050, global life expectancy is projected to rise to 78.2 years, marking a significant increase from 73.6 years in 2022 and underscoring the escalating challenge of population ageing worldwide [1]. According to data from China's seventh national population census, the proportion of individuals aged ≥65 years has increased by 4.6% over the past decade, rising from 8.9% to 13.5% [2]. As the ageing population expands, the prevalence of chronic diseases has substantially increased, driving the need for polypharmacy among older adults. The combination of increased medication use and alterations in pharmacokinetics and pharmacodynamics heightens the risk of drug-drug interactions and adverse drug reactions (ADRs) in certain disease states.

The safety of medication use in older adults is a critical issue requiring comprehensive attention. When the potential adverse effects of a medication outweigh its anticipated benefits in older adults, it is classified as a potentially inappropriate medication (PIM) [3]. Prescriptions involving PIMs are termed potentially inappropriate prescribing (PIP) [4]. PIP is a major contributor to the increased medication safety risks in this population [5]. A study conducted in Ireland involving 338 801 older patients found that PIP accounted for 9% of all medication expenditures, suggesting that reducing PIP could save over EUR 45 million annually [6]. Similarly, research in the UK involving 166 108 elderly individuals indicated that PIP contributed to an additional EUR 6 million in drug costs [7]. In Canada, the cost of PIP among older adults reached USD 419 million in 2013, with associated extra health care expenses totalling USD 1.4 billion [8]. A study that monitored mortality in 232 older patients through telephone interviews revealed a significant association between PIP and increased risk of death in Australia (adjusted odds ratio (AOR) = 1.45; 95% CI (confidence interval) = 1.05, 1.99) [9]. Moreover, a meta-analysis demonstrated that PIP in older patients was positively correlated with readmissions (AOR = 1.91; 95% CI = 1.21, 3.01), functional decline (AOR = 1.60; 95% CI = 1.28, 2.01), and ADRs (AOR = 1.26; 95% CI = 1.11, 1.43), increasing these risks by 91%, 60%, and 26%, respectively. PIP was also associated with a higher incidence of falls [10].

To standardise the identification of PIM, international tools such as the Beers and screening tool of older persons’ prescriptions/screening tool to alert doctors to the right (STOPP/START) criteria have been widely adopted. In China, a localised version of these criteria was developed in 2018 through a Delphi consensus process, incorporating data from national adverse drug reaction monitoring systems and clinical insights from 22 hospitals in Beijing. This adaptation sought to address region-specific prescribing patterns and comorbidities prevalent among Chinese older adults.

Globally, the prevalence of PIP among older outpatient populations is reported at 36.7%, with an increasing trend over the past two decades [11]. A study in Western Australia spanning 13 years (1993–2005) involving 251 305 older adults, of which 187 616 (74.7%) were identified with PIP, indicated a gradual decline in the prevalence of PIP, from 45.1% to 40.0% [12]. A similar evaluation using the 2012 Beers criteria to assess PIP prevalence in the UK for the years 2003, 2007, and 2011 also showed a decreasing trend over time [13]. However, a study conducted in Ireland examining PIP in older patients within primary care settings from 1997–2012 reported an increase in prevalence, from 32.6% in 1997 to 37.3% in 2012 [14]. In Saudi Arabia, research on PIP prevalence in outpatient prescriptions for older patients showed an increase from 57.2% in 2017 to 60.4% in 2019 [15].

Despite the existing global knowledge, no prior research has explored the temporal trends of PIP among older outpatients in China. Analysing these trends would provide valuable insights into medication usage patterns and changes over time in outpatient settings, revealing PIP safety concerns. Such information could inform targeted interventions for medications or regions with rising PIP incidence, including enhanced physician training or the implementation of decision-support systems, ultimately improving medication safety and reducing the occurrence of ADRs in older populations. It can also provide important clues and directions for clinical pharmacy research. Researchers can delve into the prevalence of PIP, such as the characteristics of the drugs, the physiological and pathological characteristics of the older adults, and doctors' prescribing habits, based on the changes in the prevalence of PIP, so as to provide a basis for the development of drugs more suitable for the elderly and the optimisation of drug treatment regimens.

## METHODS

### Study population, setting, and data source

We collected prescription data through a multicentre collaboration coordinated by the Chinese Pharmaceutical Association, encompassing 59 hospitals across six geographic regions – North, East, South, Northeast, Central, and Southwest China. The project, initiated in Beijing in 2015, expanded to include Shanghai, Guangzhou, Shenyang, Zhengzhou, and Chengdu, ensuring a diverse representation of health care settings across the country.

We conducted the study in these six cities and employed purposive sampling to select several secondary-level and tertiary-level hospitals in each city. For older outpatient prescriptions at these institutions, a purposive sampling was taken quarterly over a 10-day period, split into two five-day intervals. Hospitals with an established hospital information system (HIS) provided electronic prescription data through the HIS. In hospitals lacking HIS or where electronic prescription data were incomplete, paper prescriptions were sampled and manually entered into an electronic database. The collected data included basic information such as city name, disease diagnosis, department name, drug name, drug specifications, administration route, quantity dispensed, dosage, usage instructions, patient sex, and age. We included only prescriptions involving medications for older outpatients (aged ≥65 years) and excluded prescriptions that did not involve any medications, such as sterile water for injection or contrast agents. The prescription period spanned from 1 January 2015 to 31 December 2021. We adhered to STROBE reporting guidelines [16].

### Diagnosis and medication classification

We classified prescription diagnoses according to the International Classification of Diseases, 10th revision (ICD-10). We assigned drug categories based on the World Health Organization (WHO) Anatomical Therapeutic Chemical classification system and generic drug names. A clinical doctor and a clinical pharmacist were responsible for classifying the prescriptions using ICD-10 codes. The clinical pharmacist assigned ICD-10 codes to diagnoses that matched the ICD-10 system. For diagnoses that could not be directly matched, the clinical doctor utilised their expertise to assign appropriate codes. In cases where the clinical doctor was unable to assign a code, a consensus was reached through a discussion between two clinical doctors and one clinical pharmacist. For prescriptions missing clinical diagnoses, the research team contacted the relevant medical institutions by phone for clarification. Drug categorisation was performed independently by two clinical pharmacists. In cases of disagreement, a senior clinical pharmacist made the final decision. Each prescription was evaluated by two researchers and confirmed by a third. For prescriptions with missing drug names, the research team contacted the relevant medical institutions by phone for confirmation. Prescriptions lacking sufficient information regarding drug names and diagnoses were excluded.

### Evaluation criteria

In this study, we employed the Chinese criteria to assess the use of PIMs in outpatient prescriptions for older adults. The evaluation was carried out by two clinical pharmacists specialising in chronic diseases, who independently conducted the assessments using an information system. Upon completion of their individual evaluations, a geriatrics-specialised clinical pharmacist performed a manual review and verification of the results. The Chinese criteria categorise PIMs into two main domains – 72 medications universally cautioned against in older adults and 44 medications contraindicated in 27 specific clinical conditions, ensuring their applicability to complex comorbidities (Appendix S1–2 in the **Online Supplementary Document**). In total, there were 106 evaluation criteria. We classified a prescription as a PIP if it includes any of the 72 medications or the 44 medications related to the 27 disease states outlined in the Chinese criteria. We evaluated each PIP based on the number of PIM entries it contained, according to the aforementioned criteria. If a prescription contained one of the 106 PIMs, it was considered a single PIM-related PIP. If it contained two or more of the 106 PIMs, we classified it as multiple PIM-related PIPs.

### Outcomes

We examined several key outcomes. First, we examined the overall prevalence of PIP, providing a broad understanding of its occurrence within the studied population. Second, we assessed the prevalence of PIP in relation to the number of PIMs in each prescription. We chose this due to the increasing complexity of clinical interventions as the number of PIMs in a prescription rises. For example, a prescription containing multiple PIMs requires more complex management strategies than one with a single PIM. Finally, we considered the prevalence of PIP associated with high-risk PIMs. We selected these high-risk PIMs because they constitute the majority of detected PIMs. By targeting control measures at these high-risk PIMs, we enhanced the efficiency of PIP management, thereby reducing the overall prevalence of PIP and improving medication safety for patients.

### Statistical analysis

We calculated the annual prevalence of PIP by dividing the number of prescriptions associated with PIM at least once by the total number of prescriptions in the study, with 95% CIs. As the data distribution does not conform to the normal distribution, we evaluated trends using the average annual percent change (AAPC) calculated via a Poisson regression model [17]. We defined statistical significance by a two-tailed *P*-value <0.05, and we generated all joinpoint analyses and plots using the Joinpoint Regression program, version 5.0.2 (National Cancer Institute, Bethesda, Maryland, USA).

## RESULTS

### Prescription characteristics

Between 2015–21, we analysed data from 59 hospitals, covering 982 605 outpatient prescriptions for older adults (Appendix S3 in the **Online Supplementary Document**). Of these, 291 964 prescriptions were identified as PIP, resulting in a PIP prevalence of 29.71%. Both the total number of prescriptions and the prevalence of PIP increased from 2015, reaching their peak in 2019, before decreasing in 2021. Among the six cities studied, Shanghai reported the highest number of prescriptions and PIP occurrences, while Zhengzhou had the lowest PIP prevalence. Secondary-level hospitals issued 83 515 prescriptions (8.50%) and 23 666 PIP prescriptions (8.11%), while tertiary-level hospitals accounted for the majority, issuing 899 090 prescriptions (91.50%) and 268 298 PIP prescriptions (91.89%). A breakdown by gender revealed that male patients received 627 329 prescriptions (63.84%) and 190 619 PIP prescriptions (65.29%), while female patients received 355 276 prescriptions (36.16%) and 101 345 PIP prescriptions (34.71%). The median age of patients was 81 years (interquartile range (IQR) = 73, 87), with an age range 65–120 years. Patients aged ≥80 years comprised 56.10% of the total prescriptions (**Table 1**; Appendix S4 in the **Online Supplementary Document**). Among the 291 964 PIPs, there were 373 018 PIMs, with clopidogrel being the most commonly identified PIM, followed by estazolam, sliding-scale insulin, zolpidem, and alprazolam. These five medications represented 56.26% of all PIMs (**Table 2**).

**Table 1:** Basic characteristics of the study PIPs*

	Year							
**Characteristics**	**2015**	**2016**	**2017**	**2018**	**2019**	**2020**	**2021**	**Total**
Numbers of PIMs								
*Single PIM-related PIP*	27 143 (74.06)	34 581 (82.82)	35 782 (81.31)	38 892 (80.37)	39 254 (80.73)	30 817 (80.38)	30 219 (79.01)	236 688 (81.07)
*Multiple PIM-related PIP*	5456 (16.74)	7171 (17.18)	8227 (18.69)	9802 (19.63)	9371 (19.27)	7523 (19.62)	8026 (20.99)	55 276 (18.93)
City								
*Beijing*	11 267 (34.56)	12 029 (28.81)	11 426 (25.96)	12 454 (25.73)	11 590 (23.84)	6625 (17.28)	6637 (17.35)	72 028 (24.67)
*Chengdu*	3692 (11.33)	4537 (10.87)	4273 (9.71)	4905 (10.14)	4550 (9.36)	4216 (11.00)	3056 (7.99)	29 229 (10.01)
*Guangzhou*	2646 (8.12)	8491 (20.34)	8993 (20.43)	9857 (20.37)	9687 (19.92)	7451 (19.43)	7400 (19.35)	54 525 (18.68)
*Shanghai*	10 592 (32.49)	11 308 (27.08)	12 139 (27.58)	11 653 (24.08)	12 552 (25.81)	10 871 (28.35)	11 084 (28.98)	80 199 (27.47)
*Shenyang*	3775 (11.58)	4660 (11.16)	6118 (13.90)	8134 (16.81)	8825 (18.15)	7950 (20.74)	8449 (22.09)	47 911 (16.41)
*Zhengzhou*	627 (1.92)	727 (1.74)	1060 (2.41)	1391 (2.87)	1421 (2.92)	1227 (3.20)	1619 (4.23)	8072 (2.76)
Hospital level								
*2nd*	2984 (9.15)	3598 (8.62)	4510 (10.25)	3631 (7.50)	3117 (6.41)	2813 (7.34)	3013 (7.88)	23 666 (8.11)
*3nd*	29 615 (90.85)	38 154 (91.38)	39 499 (89.75)	44 763 (92.50)	45 508 (93.59)	35 527 (92.66)	35 232 (92.12)	268 298 (91.89)
Sex								
*Male*	21 884 (67.13)	28 101 (67.30)	29 210 (66.37)	31 760 (65.63)	31 522 (64.83)	24 107 (62.88)	24 035 (62.84)	190 619 (65.29)
*Female*	10 715 (32.87)	13 651 (32.70)	14 799 (33.63)	16 634 (34.37)	17 103 (35.17)	14 233 (37.12)	14 210 (37.16)	101 345 (34.71)
Age group in years								
65–79	12 138 (37.23)	15 512 (37.15)	15 999 (36.35)	18 194 (37.60)	18 798 (38.66)	15 730 (41.03)	16 100 (42.10)	112 471 (38.52)
≥80	20 461 (62.77)	26 240 (62.85)	28 010 (63.65)	30 200 (62.40)	29 827 (61.34)	22 610 (58.97)	22 145 (57.90)	179 493 (61.48)

PIM – potentially inappropriate medication, PIP – potentially inappropriate prescribing

*Presented as n (%).

**Table 2:** PIM used in the prescriptions

	Year							
**Characteristics**	**2015 (n = 116 037)**	**2016 (n = 138 669)**	**2017 (n = 143 232)**	**2018 (n = 154 141)**	**2019 (n = 165 481)**	**2020 (n = 133 151)**	**2021 (n = 131 894)**	**Total (n = 982 605)**
PIM (n)	42 560	51 064	54 692	60 797	64 149	50 362	49 394	373 018
Clopidogrel	9795 (8.44)	13 991 (10.09)	14 130 (9.87)	14 248 (9.24)	13 873 (8.38)	9252 (6.95)	8893 (6.74)	84 182 (8.57)
Estazolam	4724 (4.07)	6491 (4.68)	6993 (4.88)	7792 (5.06)	8127 (4.91)	7105 (5.34)	6643 (5.04)	47 875 (4.87)
Sliding-scale insulin	3998 (1.64)	4583 (1.71)	4775 (2.04)	5115 (2.40)	5541 (2.77)	4208 (2.90)	4253 (3.42)	32 473 (3.30)
Zolpidem	1899 (3.45)	2373 (3.30)	2927 (3.33)	3692 (3.32)	4586 (3.35)	3868 (3.16)	4510 (3.22)	23 855 (2.43)
Alprazolam	1988 (1.71)	2377 (1.71)	2898 (2.02)	3444 (2.23)	3597 (2.17)	3557 (2.67)	3606 (2.73)	21 467 (2.18)

PIM – potentially inappropriate medication

*Presented as n (%).

### Trends in the prevalence of PIP

**Figure 1:**
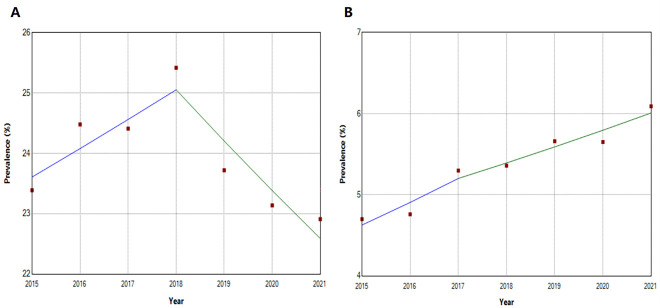
Trends in the prevalence of single and multiple PIM-related PIPs. **Panel A.** Single PIM-related PIP. **Panel B.** Multiple PIM-related PIP.

**Figure 2:**
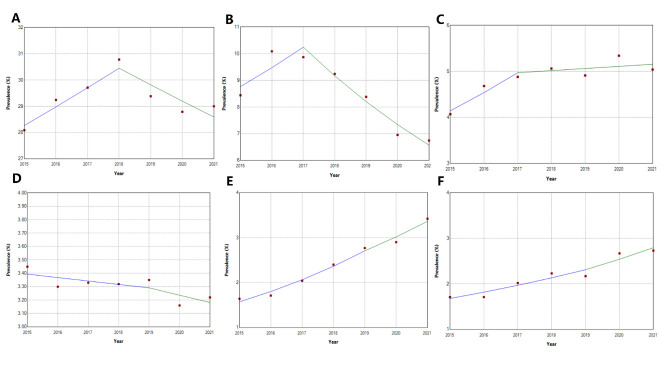
Trends in the prevalence of PIP associated with high-risk PIM. **Panel A.** Overall prevalence of PIP. **Panel B.** Clopidogrel. **Panel C.** Estazolam. **Panel D.** sliding-scale insulin. **Panel E.** Zolpidem. **Panel F.** Alprazolam.

Between 2015–21, the prevalence of PIP among older outpatients in China showed a slight increase, from 28.09% in 2015 to 29.00% in 2021, with no statistically significant overall upward trend (AAPC = 0.19; 95% CI = –1.03, 1.46). The prevalence trend of PIP varied depending on the number of PIMs. The prevalence of PIP involving a single PIM showed no statistically significant change over time (AAPC = –0.73; 95% CI = –2.42, 1.01), while the prevalence of PIP involving multiple PIMs exhibited an upward trend (AAPC = 4.45; 95% CI = 1.77, 7.20). Notably, the prevalence of PIP in Guangzhou and Shanghai increased, with the rise in Guangzhou (AAPC = 8.59; 95% CI = 4.74, 14.38) outpacing that of Shanghai (AAPC = 1.67; 95% CI = 0.55, 2.84). Furthermore, the prevalence of PIP among women showed a significant increase (AAPC = 1.94; 95% CI = 0.89, 3.12) (**Figure 1**, Panel A–B, **Figure 2**, Panel A, **Table 3**).

**Table 3:** The trend of the overall prevalence of PIP in older outpatients between 2015–21

Characteristic	Prevalence of PIP by year (%)							AAPC (95%CI)
	**2015**	**2016**	**2017**	**2018**	**2019**	**2020**	**2021**	
Single PIM-related PIP	23.39	24.48	24.41	25.42	23.72	23.14	22.91	–0.73 (–2.42, 1.01)
Multiple PIM-related PIP	4.70	4.76	5.30	5.36	5.66	5.65	6.09	4.45 (1.760, 7.204)*
City								
*Beijing*	35.68	36.09	34.97	35.53	33.40	34.14	36.01	–0.25 (–1.66, 0.88)
*Chengdu*	36.14	33.48	37.63	36.66	34.17	35.14	35.77	–0.16 (–1.85, 1.58)
*Guangzhou*	25.18	32.43	36.72	41.28	44.06	44.68	41.72	8.59 (4.74, 14.38)*
*Shanghai*	23.42	23.43	23.56	24.25	24.44	26.09	25.09	1.67 (0.55, 2.84)*
*Shenyang*	22.33	23.66	23.66	24.03	21.67	19.69	21.60	–1.89 (–4.40, 0.58)
*Zhengzhou*	38.94	38.36	40.81	43.57	41.67	40.35	41.58	1.15 (–1.52, 4.13)
Hospital level								
*2nd*	25.33	32.13	29.05	26.77	25.59	28.68	29.21	–0.03 (–5.82, 5.83)
*3nd*	28.17	30.06	30.93	31.84	29.69	28.80	28.98	–0.18 (–1.36, 1.06)
Sex								
*Male*	29.39	31.53	32.15	32.19	29.82	28.31	28.83	–0.85 (–3.64, 1.57)
*Female*	25.78	27.56	28.26	29.98	28.60	29.65	29.28	1.94 (0.89, 3.12)*
Age group in years								
*65–79*	24.66	26.45	26.74	27.82	25.81	25.56	25.20	–0.16 (–1.29, 1.02)
*≥80*	30.63	32.79	33.58	34.03	32.20	31.58	32.57	0.53 (–1.56, 2.47)
Total	28.09	29.24	29.71	30.78	29.38	28.79	29.00	0.19 (–1.03, 1.46)

PIM – potentially inappropriate medication, PIP – potentially inappropriate prescribing

*The difference was statistically significant.

### Trends in the prevalence of PIP associated with high-risk PIMs

The prevalence of PIP for clopidogrel demonstrated a significant downward trend, decreasing from 8.44% in 2015 to 6.74% in 2021 (AAPC = –4.70; 95% CI = –10.16, –0.43). This decline was observed in Beijing, Shanghai, and Zhengzhou, with the fastest decrease occurring in Beijing (AAPC = –6.71; 95% CI = –9.46, –4.18), followed by Shanghai (AAPC = –5.79; 95% CI = –8.94, –3.27) and Zhengzhou (AAPC = 5.30; 95% CI = 9.75, –1.08). Across different age groups, the prevalence of PIP for clopidogrel also declined, with a steeper reduction seen in patients aged 65–79 years (AAPC = –5.74; 95% CI = –10.57, –1.19) compared to those aged ≥80 years (AAPC = –3.83; 95% CI = –6.60, 1.46) (**Figure 2**, Panel B; Appendix S5 in the **Online Supplementary Document**).

The prevalence of PIP for estazolam showed a significant upward trend, increasing from 4.07% in 2015 to 5.04% in 2021 (AAPC = 3.71; 95% CI = 1.57, 5.77). This rise was observed in Beijing, Guangzhou, Shanghai, and Zhengzhou, with Guangzhou exhibiting the fastest increase (AAPC = 24.81; 95% CI = 15.72, 39.85). Across age groups, the prevalence of PIP for estazolam also rose, with the steepest increase in patients aged 65–79 years (AAPC = 4.45; 95% CI = 1.28, 7.97) compared to those aged ≥80 years (AAPC = 3.58; 95% CI = 1.47, 5.34) (**Figure 2**, Panel C; Appendix S6 in the **Online Supplementary Document**).

Similarly, the prevalence of PIP for zolpidem increased significantly, rising from 1.64% in 2015 to 3.42% in 2021 (AAPC = 13.51; 95% CI = 8.72, 18.50). This upward trend was observed in all cities, with Guangzhou exhibiting the fastest growth rate (AAPC = 38.70; 95% CI = 31.14, 58.71). The prevalence of PIP for zolpidem also rose across different health care levels, genders, and age groups. Notably, secondary health care institutions (AAPC = 36.10; 95% CI = 32.40, 42.40), females (AAPC = 15.97; 95% CI = 12.29, 20.82), and individuals aged 65–79 years (AAPC = 14.46; 95% CI = 8.35, 22.42) experienced more rapid increases (**Figure 2**, Panel E; Appendix S7 in the **Online Supplementary Document**).

In contrast, the prevalence of PIP for sliding-scale insulin did not show a significant change overall (AAPC = –1.07; 95% CI = –3.02, 0.92). However, a downward trend was observed in Chengdu, Shanghai, and Shenyang, with Chengdu experiencing the fastest decline (AAPC = –7.63; 95% CI = –9.03, –6.37), followed by Shenyang (AAPC = –3.78; 95% CI = –6.37, –1.20) and Shanghai (AAPC = –1.98; 95% CI = –3.77, –0.19) (**Figure 2**, Panel D; Appendix S8 in the **Online Supplementary Document**).

The prevalence of PIP for alprazolam increased notably, rising from 1.71% in 2015 to 2.73% in 2021 (AAPC = 8.86%; 95% CI = 3.8614.10). Both Shanghai and Zhengzhou exhibited upward trends, with Zhengzhou experiencing the most rapid increase (AAPC = 26.18; 95% CI = 14.68, 48.40), followed by Shanghai (AAPC = 15.28; 95% CI = 7.64, 25.92). This trend was consistent across different health care levels, genders, and age groups, with secondary health care institutions (AAPC = 14.78; 95% CI = 8.21, 23.35), males (AAPC = 9.42; 95% CI = 6.61, 12.85), and individuals aged 65–79 years (AAPC = 10.41; 95% CI = 7.81, 13.05) experiencing more rapid increases (**Figure 2**, Panel F; Appendix S9 in the **Online Supplementary Document**).

## DISCUSSION

To the best of our knowledge, this study represents the first attempt to assess trends in the prevalence of PIP among older adults in outpatient services across China. Using a joinpoint regression model, we analysed the trends in the prevalence of PIP in 982 605 older outpatient prescriptions from six Chinese cities between 2015–21. The findings indicate no statistically significant overall trend in the prevalence of PIP during this period. In contrast, a study in Italy examining the prevalence of PIP among older patients in Pistoia between 2012–18 revealed a decline, from 43.27% in 2012 to 33.24% in 2018 [18]. Similarly, a study of national data from France’s health insurance system reported a significant decrease in the prevalence of PIP among older outpatients, from 49.6% in 2011 to 39.6% in 2019 (AAPC = –1.19; 95% CI = –1.35, –1.04) [19]. Conversely, a study conducted in Ireland found an increase in the prevalence of PIP, from 42.3% in 2012 to 51.0% in 2015, based on data from 44 health care institutions [20]. These discrepancies in trends across countries may reflect differences in medical advancements, improvements in clinical practice, and growing awareness of PIP among health care professionals. The introduction of PIM criteria has been instrumental in raising clinicians' awareness of PIP. Research has shown that the implementation of the STOPP/START criteria has positively impacted PIP management, reducing ADRs and improving medication adherence in older patients [21]. However, a survey of 597 clinicians in China revealed that more than half (54.9%) were unaware of the Chinese PIM criteria [22], highlighting the need for increased clinical promotion of these criteria to improve their application in managing PIP among older outpatients. There should be regulatory enforcement, clinical decision support systems, or continuing medical education requirements.

We also revealed an increasing trend in the prevalence of PIP involving multiple PIMs among older outpatient prescriptions. A two-year follow-up study on older patients with prescriptions containing single or multiple PIMs found that those with multiple PIMs were at a higher risk of ADRs (incidence rate ratio (IRR) = 1.29; 95% CI = 1.03, 1.60), had a lower quality of life (β = –0.11; 95% CI = 0.16, –0.06), and exhibited a higher rate of emergency department visits (AOR = 1.85; 95% CI = 1.06, 3.24) [23]. The likelihood of multiple PIMs increases with the number of medications prescribed [24]. An analysis of polypharmacy trends among elderly outpatients in Asia between 2013–16 showed rising prevalence rates in Hong Kong (AAPC = 3.7; 95% CI = 2.1, 3.3), Taiwan (AAPC = 1.0; 95% CI = 0.7, 1.3), and South Korea (AAPC = 1.7; 95% CI = 1.4, 2.3) [25]. Polypharmacy is hypothesised to be a key driver of the increase in PIP involving multiple PIMs. It is recommended that future efforts focus on medication reviews in outpatient settings, with greater collaboration between physicians and pharmacists, to reduce the prevalence of PIP and polypharmacy.

The results demonstrated an upward trajectory in the prevalence of PIP among older outpatients in Guangzhou and Shanghai between 2015–21, while trends in other cities showed less significance. First-tier cities like Shanghai and Guangzhou, with more abundant health care resources, tend to attract older patients with complex or severe conditions, thereby increasing the risk of PIP due to the necessity for multiple medications. Interestingly, the prevalence trend in Beijing, also a first-tier city, was less pronounced. This may be attributed to the development of the Chinese criteria for PIM primarily under the leadership of medical institutions in Beijing. Consequently, local clinicians may possess a more nuanced understanding of PIP risks and demonstrate greater vigilance in addressing PIM issues during prescribing practices.

Among the medications identified in this study, the top five included three benzodiazepines. The high prescription frequency of benzodiazepines can primarily be attributed to the widespread issue of insomnia among older patients, with over 80% of older patients being prescribed insomnia medications. However, a notable concern is that many of these prescribed benzodiazepines and other sedative-hypnotic drugs are inappropriate for older patients [26]. The data revealed a decreasing trend in the prevalence of PIP related to clopidogrel between 2015–21, likely due to the introduction of newer medications, such as ticagrelor, which provide more effective antiplatelet action and enhanced safety for older patients [27]. Nevertheless, the use of sedative-hypnotic drugs, including estazolam, zolpidem, and alprazolam, has been on the rise. The prevalence of insomnia among older patients in China ranges from 43.90% to 53.89% and has been consistently increasing, which likely contributes to the growing consumption of these sedative-hypnotic medications. According to the Chinese Sleep Study Report, the sleep index in South China is relatively low, and Guangzhou belongs to this region. Therefore, benzodiazepines are rising so sharply, especially in cities like Guangzhou. The increased use of sedative-hypnotics is tied to pharmaceutical marketing, insufficient geriatric training, or systemic pressures in outpatient care.

Between 2015–21, no statistically significant changes were observed in the overall rate of PIP across various health care levels, age groups, or among male patients. However, the prevalence of PIP among female patients showed a tendency to increase. Previous studies have indicated that the prevalence of comorbidities among older women in China surpasses that of men and continues to rise, which may account for the increasing PIP rates among female outpatients [28]. According to the relevant survey data of the Chinese Geriatrics Society, compared with men, women generally exhibit stronger health awareness, which makes them more inclined to actively seek medical assistance. This characteristic is particularly evident among the older population. Due to frequent medical visits, women have significantly increased opportunities to be exposed to various drugs, thus increasing the risk of drug exposure. In tertiary hospitals, the prevalence of PIP due to estazolam, zolpidem, and alprazolam rose, while that associated with clopidogrel and insulin declined. In secondary hospitals, an increase in the prevalence of PIP related to zolpidem and alprazolam was also observed. This pattern may be attributed to the more rapid knowledge updates among clinicians in tertiary institutions, influencing their prescribing practices. As clinical knowledge advances, physicians are more likely to select safer, non-PIM medications. The growing use of sedative-hypnotic drugs in both secondary and tertiary hospitals underscores the widespread prescription of these medications to older outpatients. Thus, targeted interventions are recommended to address the use of these drugs [29].

Deprescribing refers to the planning and management process of reducing the dosage of a medication or discontinuing a medication that may cause harm to older patients or from which older patients no longer benefit. Its goal is to reduce the burden and harm of medications while maintaining or improving the therapeutic effect [30]. The core of deprescribing is to identify PIMs for older patients, develop a PIM reduction plan together with elderly patients, and conduct follow-up on the effect of deprescribing. A study analysed 12 systematic reviews, and the research results showed that deprescribing can reduce the risk of PIMs and PIP in older patients [31].

## CONCLUSIONS

Between 2015–21, the prevalence of PIP involving multiple PIMs among older outpatients in China exhibited an upward trend. Among the five high-risk PIMs, the prevalence of PIP for estazolam, zolpidem, and alprazolam increased, while the prevalence of clopidogrel decreased.

## Additional material


Online Supplementary Document
