# Supplementary material for: Trends of potentially inappropriate prescribing among older outpatients in China between 2015–21
Source: J Glob Health. 2025 Jun 20;15:04190. doi: 10.7189/jogh.15.04190 (PMC12178595; doi:10.7189/jogh.15.04190)
Supplement: Online Supplementary Document [file jogh-15-04190-s001.pdf]

Supplement to: Tian F, Chen Z, Zhang Y, Feng Q. Trends of potentially inappropriate prescribing among older outpatients in China between 2015–21. J Glob Health. 2025;15:04190.

Contents:

Appendix 1. Classification of 72 PIMs based on pharmacological

Appendix 2. Based on 44 PIM classifications across 27 disease states

Appendix 3. Basic information of the hospitals included in the study

Appendix 4. Basic characteristics of the study prescriptions

Appendix 5. The trend of the prevalence of PIP for clopidogrel in older outpatients from 2015 to 2021

Appendix 6. The trend of the prevalence of PIP for estazolam in older outpatients from 2015 to 2021

Appendix 7. The trend of the prevalence of PIP for zolpidem in older outpatients from 2015 to 2021

Appendix 8. The trend of the prevalence of PIP for sliding-scale insulin in older outpatients from 2015 to 2021

Appendix 9. The trend of the prevalence of PIP for alprazolam in older outpatients from 2015 to 2021

Appendix 1. Classification of 72 PIMs based on pharmacological

Pharmacological	Drug name
Nervous system drugs	Lorazepam, alprazolam, phenazopyridine, D dihydroergotoxine, estazolam, nicergoline, zolpidem, clozapine, nitrazepam, barbiturates, phenobarbital, clonazepam, diazepam, phenytoin, pentoxifylline
Psychiatric drugs	Fluoxetine, risperidone, olanzapine, quetiapine, amitriptyline, chlorpromazine, doxepin, maprotiline, clozapine, phenazine, fluphenazine, haloperidol, aripiprazole, fluvoxamine, sulpiride
Antipyretic, analgesic, anti-inflammatory and antirheumatic drugs	Nabumetone, diclofenac, ibuprofen, indomethacin, ≥2 NSAIDs in combination, prednisone, piroxicam, naproxen, ketoprofen, etoricoxib
Cardiovascular drugs	Rifampicin (>0.1mg/d), later referred to as rifampicin, doxazosin, digoxin (>0.125mg/d), later referred to as digoxin, amiodarone, colestipol, procainamide, nifedipine (immediate-release dosage form), later referred to as nifedipine
Endocrine system drugs	Insulin (sliding scale, sliding scale), growth hormone, glibenclamide, megestrol
Blood system drugs	Warfarin, clopidogrel, ticlopidine
Urinary system drugs	Spironolactone (>25mg/d) later referred to as spironolactone, tolterodine
Respiratory system drugs	theophylline
Anti-infective drugs	Gatifloxacin, aminoglycoside antibiotics, vancomycin, clindamycin
Digestive system drugs	Scopolamine analogues, belladonna alkaloids, cimetidine
Anaesthetics and anaesthetic drugs	Pethidine, morphine, morphine extended-release tablets, tramadol
Bone muscle relaxants	Baclofen, chlorzoxazone

Appendix 2. Based on 44 PIM classifications across 27 disease states

**Disease state**	**Drug name**
Epilepsy or seizures	Antipsychotics, thioridazine, bupropion, maprotiline
Delirium	Benzodiazepines, chlorpromazine, tricyclic antidepressants, glucocorticoids, anticholinergics, thioridazine
Dementia or cognitive impairment	Benzodiazepines
Insomnia	Phenylephrine, pemoline, triazolam
Parkinson's disease	Antipsychotics, metoclopramide, promethazine, haloperidol
Cognitive impairment	Anticholinergics
Stroke prevention	Dipyridamole
Depression	Risperdal
Heart failure	NSAIDs, diltiazem, verapamil, pioglitazone, rosiglitazone, cilostazol
Syncope	Chlorpromazine, olanzapine, doxazosin, terazosin, cholinesterase inhibitors, thioridazine
Postural hypotension	Chlorpromazine
Hypertension	NSAIDs, rifampicin
Coagulation disorders or on anticoagulant therapy	Ticlopidine, clopidogrel, NSAIDs
Chronic constipation	Cyproheptadine, oxybutynin
Chronic renal insufficiency	NSAIDs
Chronic kidney disease stage IV/V	Amphotericin
Urinary incontinence	Estrogen, doxazosin, prazosin, terazosin
Lower urinary tract symptoms, prostate enlargement	Anticholinergics
Peptic ulcer	NSAIDs, glucocorticoids
Chronic constipation	Antipsychotics, tricyclic antidepressants, propantheline bromide, tolterodine, anticholinergics, chlorpheniramine, clemastine, diphenhydramine
Chronic obstructive pulmonary disease	Benzodiazepines
Sleep apnoea syndrome	Benzodiazepines
Osteoporosis	Glucocorticoids
Diabetes mellitus	Glucocorticoids (long-term use)
Falls or fracture history	Benzodiazepines, zaleplon, antipsychotics, tricyclic antidepressants
Glaucoma	Tricyclic antidepressants, anticholinergics<br>Pain Pethidine
Pain	Pethidine
Gout	Thiazide diuretics
Falls or fracture history	Dexzopiclone

Appendix 3. Basic information of the hospitals included in the study

**City**	**Hospital code**	**No**	
		**2nd**	**3nd**
Beijing	211, 214, 301, 302, 304, 310, 313, 318, 319	2	7
Chengdu	211, 212, 213, 305, 306, 307, 308, 310, 312	3	6
Guangzhou	301, 302, 305, 306, 309, 314	0	6
Shanghai	208, 218, 220, 223, 227, 302, 305, 306, 309, 310, 311, 312, 314, 321, 322, 326, 328	5	12
Shenyang	301, 303, 304, 305, 306, 308, 309, 310, 311, 312, 313	0	11
Zhengzhou	207, 304, 306, 308, 313, 314, 316	1	6
Total		11	48

Appendix 4. Basic characteristics of the study prescriptions

Characteristic	2015	2016	2017	2018	2019	2020	2021	Total
City								
Beijing	31,582 (27.22)	32,519 (23.45)	31,725 (22.15)	34,984 (22.70)	34,696 (20.97)	19,407 (14.58)	18,429 (13.97)	203,342 (20.69)
Chengdu	10,216 (8.80)	12,964 (9.35)	10,978 (7.66)	12,928 (8.39)	13,313 (8.05)	11,998 (9.01)	8,543 (6.48)	80,940 (8.24)
Guangzhou	10,507 (9.05)	25,814 (18.62)	24,179 (16.88)	23,410 (15.19)	21,986 (13.29)	16,678 (12.53)	17,737 (13.45)	140,311 (14.28)
Shanghai	45,219 (38.97)	46,415 (33.47)	48,560 (33.90)	46,845 (30.39)	51,354 (31.03)	41,660 (31.29)	44,176 (33.49)	324,229 (33.00)
Shenyang	16,903 (14.57)	19,135 (13.80)	25,271 (17.64)	32,839 (21.30)	40,722 (24.61)	40,367 (30.32)	39,115 (29.66)	214,352 (21.81)
Zhengzhou	1,610 (1.39)	1,822 (1.31)	2,519 (1.76)	3,135 (2.03)	3,410 (2.06)	3,041 (2.28)	3,894 (2.95)	19,431 (1.98)
Hospital level								
2nd	10,435 (8.99)	11,687 (8.43)	15,527 (10.84)	13,564 (8.80)	12,180 (7.36)	9,808 (7.37)	10,314 (7.82)	83,515 (8.50)
3nd	105,602 (91.01)	126,982 (91.57)	127,705 (89.16)	140,577 (91.20)	153,301 (92.64)	123,343 (92.63)	121,580 (92.18)	899,090 (91.50)
Sex								
Male	74,466 (64.17)	89,131 (64.28)	90,863 (63.44)	98,663 (64.01)	105,690 (63.87)	85,147 (63.95)	83,369 (63.21)	627,329 (63.84)
Female	41,571 (35.83)	49,538 (35.72)	52,369 (36.56)	55,478 (35.99)	59,791 (36.13)	48,004 (36.05)	48,525 (36.79)	355,276 (36.16)
Age group, years								
65-79	49,231 (42.43)	58,638 (42.29)	59,823 (41.77)	65,406 (42.43)	72,837 (44.02)	61,544 (46.22)	63,893 (48.44)	431,372 (43.90)
≥80	66,806 (57.57)	80,031 (57.71)	83,409 (58.23)	88,735 (57.57)	92,644 (55.98)	71,607 (53.78)	68,001 (51.57)	551,233 (56.10)
Total	116,037	138,669	143,232	154,141	165,481	133,151	131,894	982,605

Appendix 5. The trend of the prevalence of PIP for clopidogrel in older outpatients from 2015 to 2021

Characteristic	prevalence of PIP for clopidogrel (%)							AAPC (95%CI)
	2015	2016	2017	2018	2019	2020	2021	
City								
Beijing	11.99	12.45	11.80	10.93	10.02	8.05	8.66	-6.71 (-9.46~-4.18)*
Chengdu	13.43	12.09	14.35	13.82	12.06	12.09	14.39	0.21 (-5.52~6.27)
Guangzhou	10.09	15.97	17.25	18.16	18.24	15.20	12.54	1.60 (-3.38~7.25)
Shanghai	6.72	7.41	7.18	6.12	5.55	5.17	5.17	-5.79 (-8.94~-3.27)*
Shenyang	2.07	3.23	3.45	3.66	4.09	3.26	3.42	7.48 (-1.54~17.27)
Zhengzhou	11.80	10.59	11.08	10.02	7.86	7.73	9.60	-5.30 (-9.75~-1.08)*
Hospital level								
2nd	7.46	11.39	9.64	8.10	7.41	6.63	6.87	-6.33 (-12.92~0.15)
3nd	8.47	10.06	9.89	9.35	8.46	6.97	6.86	-4.48 (-10.12~-0.09)*
Sex								
Male	9.23	11.18	10.79	10.01	8.94	7.19	6.65	-6.14 (-12.34~-1.61)*
Female	7.04	8.13	8.26	7.89	7.40	6.53	7.22	-1.11 (-3.86~1.32)
Age group, years								
65-79	7.49	9.06	8.51	7.76	6.70	5.53	6.45	-5.74 (-10.57~-1.19)*
≥80	9.15	10.84	10.84	10.34	9.70	8.17	7.24	-3.83 (-6.60~-1.46)*
Total	8.44	10.09	9.87	9.24	8.38	6.95	6.74	-4.70 (-10.16~-0.43)*

*The difference was statistically significant

Appendix 6. The trend of the prevalence of PIP for estazolam in older outpatients from 2015 to 2021

Characteristic	prevalence of PIP for estazolam (%)							AAPC (95%CI)
	2015	2016	2017	2018	2019	2020	2021	
City								
Beijing	7.06	7.73	7.76	7.56	7.53	8.72	9.64	4.64 (2.66~6.35)*
Chengdu	7.71	7.46	10.08	10.46	10.40	11.01	7.33	1.28 (-8.97~16.31)
Guangzhou	2.56	4.94	5.87	7.57	8.41	10.21	10.51	24.81 (15.72~39.85)*
Shanghai	2.84	3.34	3.57	3.52	3.66	4.84	4.40	7.81 (2.36~14.27)*
Shenyang	0.00	0.03	0.02	0.02	0.01	0.03	0.03	-0.44 (-19.78~18.64)
Zhengzhou	9.44	9.88	10.52	11.71	11.67	11.90	11.97	4.35 (3.40~5.45)*
Hospital level								
2nd	3.76	5.04	5.07	4.31	4.88	6.77	6.13	7.49 (-2.17~19.47)
3nd	4.08	4.67	4.86	5.13	4.91	5.22	4.98	3.44 (1.50~5.34)*
Sex								
Male	3.97	4.44	4.70	4.79	4.66	4.81	4.79	2.97 (2.06~3.92)*
Female	4.26	5.11	5.19	5.52	5.36	6.26	5.55	4.36 (-0.15~9.50)
Age group, years								
65-79	3.36	3.87	3.96	4.22	4.08	4.38	4.64	4.45 (1.27~7.97)*
≥80	4.59	5.27	5.54	5.67	5.57	6.16	5.48	3.58 (1.47~5.34)*
Total	4.07	4.68	4.88	5.06	4.91	5.34	5.04	3.71 (1.57~5.77)*

*The difference was statistically significant

Appendix 7. The trend of the prevalence of PIP for zolpidem in older outpatients from 2015 to 2021

**Characteristic**	**prevalence of PIP for zolpidem (%)**							**AAPC (95%CI)**
	**2015**	**2016**	**2017**	**2018**	**2019**	**2020**	**2021**	
City								
Beijing	2.72	2.88	3.22	3.23	3.58	4.34	4.87	10.47 (9.09~11.78)*
Chengdu	0.44	0.42	0.65	0.53	0.54	0.54	0.85	9.15 (1.13~19.00)*
Guangzhou	0.73	1.80	2.83	4.14	5.27	6.33	6.44	38.70 (31.14~58.71)*
Shanghai	1.80	1.69	1.95	2.50	3.29	3.58	4.58	17.81 (14.54~24.74)*
Shenyang	0.60	0.66	0.77	1.01	1.01	0.97	1.12	11.12 (7.40~16.46)*
Zhengzhou	0.25	0.27	0.20	0.57	0.38	0.72	0.69	21.59 (1.28~57.63)*
Hospital level								
2nd	0.46	0.74	1.11	1.42	1.95	2.15	3.04	36.10 (32.40~42.40)*
3nd	1.67	1.73	2.16	2.49	2.84	2.96	3.53	13.54 (11.60~15.94)*
Sex								
Male	1.60	1.70	2.01	2.27	2.54	2.62	3.27	12.38 (10.45~14.60)*
Female	1.71	1.74	2.11	2.62	3.18	3.40	3.87	15.97 (12.29~20.82)*
Age group, years								
65-79	1.53	1.44	1.69	2.08	2.47	2.56	3.19	14.46 (8.35~22.42)*
≥80	1.72	1.91	2.29	2.63	3.01	3.20	3.77	13.79 (11.11~17.16)*
Total	1.64	1.71	2.04	2.40	2.77	2.90	3.42	13.51 (8.72~18.50)*

*The difference was statistically significant

Appendix 8. The trend of the prevalence of PIP for sliding-scale insulin in older outpatients from 2015 to 2021

**Characteristic**	**prevalence of PIP for sliding-scale insulin (%)**							**AAPC (95%CI)**
	**2015**	**2016**	**2017**	**2018**	**2019**	**2020**	**2021**	
City								
Beijing	3.33	3.47	3.40	3.72	3.54	3.20	3.39	-0.22 (-1.83~1.44)
Chengdu	7.04	6.93	5.82	5.11	4.78	4.63	4.52	-7.63 (-9.03~-6.37)*
Guangzhou	1.86	1.81	2.19	2.22	3.27	3.31	2.88	10.10 (3.15~21.49)*
Shanghai	2.53	2.39	2.47	2.33	2.35	2.13	2.30	-1.98 (-3.77~-0.16)*
Shenyang	4.48	4.30	4.44	4.03	3.73	3.48	3.80	-3.78 (-6.37~-1.20)*
Zhengzhou	8.14	8.45	8.14	6.99	6.83	6.25	7.47	-3.54 (-10.57~3.41)
Hospital level								
2nd	2.87	3.66	2.83	2.58	2.94	3.19	3.43	0.86 (-5.39~7.57)
3nd	3.46	3.30	3.40	3.39	3.38	3.16	3.26	-0.96 (-1.84~-0.10)*
Sex								
Male	3.57	3.53	3.63	3.57	3.57	3.37	3.40	-0.91 (-1.97~0.18)
Female	3.23	2.90	2.83	2.87	2.95	2.79	3.05	-1.11 (-2.19~0.27)
Age group, years								
65-79	3.56	3.35	3.29	3.37	3.31	3.17	3.22	-1.46 (-2.68~-0.25)*
≥80	3.36	3.27	3.37	3.28	3.38	3.15	3.32	-0.37 (-1.44~0.73)
Total	3.45	3.30	3.33	3.32	3.35	3.16	3.22	-1.07 (-3.02~0.92)

*The difference was statistically significant

Appendix 9. The trend of the prevalence of PIP for alprazolam in older outpatients from 2015 to 2021

**Characteristic**	**prevalence of PIP for alprazolam (%)**							**AAPC (95%CI)**
	**2015**	**2016**	**2017**	**2018**	**2019**	**2020**	**2021**	
City								
Beijing	0.41	0.39	0.39	0.39	0.33	0.24	0.31	-6.32 (-10.85~-2.00)*
Chengdu	4.44	3.02	4.56	4.70	4.51	4.49	4.93	3.24 (-2.32~9.66)
Guangzhou	4.97	3.78	4.64	5.25	5.06	6.62	6.51	7.36 (-1.21~18.46)
Shanghai	1.57	1.40	1.60	2.17	2.48	3.05	3.06	15.28 (7.64~25.92)*
Shenyang	0.76	0.91	0.85	0.80	0.59	0.74	0.96	0.26 (-6.77~7.81)
Zhengzhou	2.80	3.07	6.31	6.09	7.51	9.77	9.42	26.18 (14.68~48.40)*
Hospital level								
2nd	0.89	1.95	1.78	2.00	2.06	2.77	3.01	14.78 (8.21~23.35)*
3nd	1.74	1.71	2.05	2.26	2.18	2.66	2.81	8.92 (6.77~11.35)*
Sex								
Male	1.42	1.47	1.73	1.81	1.78	2.18	2.46	9.42 (6.61~12.85)*
Female	2.24	2.16	2.54	2.99	2.86	3.54	3.45	8.90 (3.89~15.12)*
Age group, years								
65-79	1.66	1.55	1.81	1.98	1.96	2.44	2.87	10.41 (7.81~13.05)*
≥80	1.75	1.84	2.17	2.42	2.34	2.87	2.78	8.64 (4.23~13.93)*
Total	1.71	1.71	2.02	2.23	2.17	2.67	2.73	8.86 (3.86~14.10)*

*The difference was statistically significant
